# Designing an explainable algorithm based on XGBoost and genetic algorithm for predicting hospitalization needs of COVID-19 patients

**DOI:** 10.1038/s41598-026-40120-6

**Published:** 2026-02-23

**Authors:** Azadeh Abkar, Mahdi Mehrabi, Amin Golabpour, Mohammad Amin Shayegan

**Affiliations:** 1 Department of Computer Engineering, Shi.C., Islamic Azad University, Shiraz, Iran; 2 School of Allied Medical Sciences, Shahroud University of Medical Sciences, Shahroud, Iran

**Keywords:** Computational biology and bioinformatics, Diseases, Health care, Mathematics and computing, Medical research

## Abstract

Timely identification of COVID-19 outpatients who are at risk of hospitalization is critical for preventing clinical deterioration and optimizing healthcare resources. Although machine-learning models have demonstrated high predictive accuracy, their limited interpretability often hinders clinical adoption. This study aims to develop a hybrid explainable framework that combines the predictive strength of XGBoost with clinically interpretable rule-based explanations to support decision-making in real clinical settings. A retrospective dataset of 1278 COVID-19 patients was analyzed after applying strict inclusion and exclusion criteria. Twenty-seven clinical, laboratory, and demographic variables were preprocessed using outlier detection, multiple imputation by chained equations, and stratified train–test splitting validated through a Kolmogorov–Smirnov test. XGBoost was trained and benchmarked against logistic regression, random forest, LightGBM, and a neural network. For interpretability, candidate rules were extracted from a constrained Random Forest and optimized via a genetic algorithm (GA) using accuracy–support multi-objective fitness. Clinical validation of rules was performed by ten physicians using the Content Validity Index (CVI; threshold ≥ 0.85). XGBoost achieved superior predictive performance with an AUC of 0.85, sensitivity of 73.5%, specificity of 88.7%, AUPRC of 0.72, and a Brier Score of 0.085. Baseline models demonstrated lower discrimination and calibration. Fairness evaluation indicated stable model behavior across demographic and comorbidity subgroups. Sensitivity analysis identified SpO_2_, CRP, age, D-dimer, ferritin, and lymphocyte percentage as the most influential predictors. From 400 initial rules, 80 were selected and refined, and 40 clinically valid rules were finalized through expert review. The explainability framework expands upon the classical Decision Tree Surrogate method, producing global IF–THEN rules that outperform local attribution tools such as SHAP and LIME in practical interpretability. The proposed hybrid system successfully integrates high-accuracy machine-learning predictions with clinically validated, interpretable rules, offering a transparent decision-support tool for hospitalization risk assessment in COVID-19 outpatients. Its modular design enables rapid adaptation to future infectious disease outbreaks, supporting broader clinical deployment and improved triage decision-making.

## Introduction

The Coronavirus disease 2019 (COVID-19) emerged as a major public health emergency that placed unprecedented pressure on healthcare systems worldwide. According to the World Health Organization, by mid-2021 more then 700 million confirmed cases and nearly 7 million deaths had been reported globally^1^. Early identification of patients who truly require hospitalization is essential, as timely admission significantly reduces the risk of severe complications, including acute respiratory distress syndrome (ARDS), multi-organ failure, and increased mortality. Research shows that, on average, 10–20% of COVID-19 patients require hospital admission^2^. Although this proportion varies across different variants of the virus. For example, hospitalization rates reached 30% during the Delta wave in some European countries^3^, whereas in Iran the rate during the same period was approximately 15%^4^. These differences reflect variations in population age structures, testing strategies, and thresholds for hospital admission, emphasizing the need for localized predictive tools to support clinical decision-making. Delayed hospitalization has also been identified as a major contributor to adverse outcomes; studies indicate that patients with severe symptoms who are not admitted within 48 h have markedly lower survival rates^5–9^. Therefore, medical systems must implement reliable mechanisms to ensure timely hospitalization for high-risk individuals.

Artificial intelligence (AI) has demonstrated strong potential in predicting hospitalization needs by analyzing clinical and paraclinical data. For effective clinical integration, such systems must follow the principles of human-centered AI: models should provide transparent reasoning, training datasets must be representative to minimize bias, and final decision-making must remain under clinical supervision^10–12^. Recent machine-learning studies, including those by Salehnaseb et al.^13^, Cisterna Garcia et al.^15^, and Bayat et al.^17^, report promising accuracy using models such as XGBoost and hybrid frameworks that incorporate demographic and laboratory parameters. However, despite improvements in predictive performance, many existing approaches rely on black-box algorithms that do not provide clinically meaningful explanations, limiting their acceptance among healthcare providers.

Artificial intelligence (AI) has demonstrated strong potential in predicting hospitalization needs by analyzing clinical and paraclinical data. The development of such systems must follow the principles of human-centered artificial intelligence to assist physicians in making optimal therapeutic decisions. To achieve this, several key requirements must be met: clinicians must be able to understand the reasoning behind model outputs^10^. Training datasets must be sufficiently comprehensive and representative to minimize unintended biases^11^, and AI systems should support decision-making while leaving final judgment to medical experts^12^. Effective implementation therefore depends on close collaboration between data scientists and clinicians to ensure that analytical accuracy aligns with practical clinical needs. Deploying machine learning models to forecast outpatient hospitalization during the COVID-19 pandemic has shown substantial promise in optimizing healthcare resource allocation. For example, Salehnasab et al. applied twenty predictive algorithms to clinical data and found that the XGBoost classifier achieved the highest performance with an AUC of 0.81^13^. Similar studies have reported that advanced age, reduced oxygen saturation, persistent fever, and underlying comorbidities are major predictive factors for hospitalization^14^. In Spain, Cisterna-García et al. analyzed data from more than 86,000 patients and developed a hybrid model combining Logistic Regression, Random Forest, and the IPIP framework, achieving an AUC of 0.75 for hospitalization prediction and even higher accuracy for mortality prediction; notably, this model requires only basic demographic and chronic illness variables, making it suitable for emergency and resource-limited settings^15^. In another study, De Diego-Castell et al. assessed the influence of obesity, kidney disease, depression, and polypharmacy on hospitalization risk, highlighting the importance of lifestyle and comorbidity factors^16^. Additionally, Bayat et al. compressed laboratory data using XGBoost to predict hospitalization with high accuracy, while Wang et al. reported a similar approach yielding an AUC of 83%^17,18^. Both studies identified age, oxygen saturation, C-reactive protein (CRP), and lymphocyte count as critical predictors of hospitalization. Overall, recent research suggests that predictive accuracy in outpatient settings has improved significantly as machine learning models increasingly combine optimized hyperparameters with diverse clinical and lifestyle variables, rather than relying on a single algorithmic approach; in this regard, the contribution of Salehnasab et al. is particularly notable^13^.

This limitation highlights a critical research gap: the lack of models that simultaneously achieve high predictive accuracy and offer interpretable, clinically verifiable decision rules. To address this need, the present study proposes a hybrid explainable framework that integrates a high-performance black-box model (XGBoost) with a set of interpretable rules generated through Random Forest and optimized via a Genetic Algorithm. In this framework, Random Forest is not used as a primary classifier but rather as a rule-extraction tool, as its numerous shallow decision trees can be systematically transformed into human-readable IF–THEN rules. A complementary probabilistic rule-selection mechanism is then applied to refine and validate the extracted rules. This approach aims to enhance model transparency and foster greater clinical trust in machine-learning-based decision support systems for predicting hospitalization needs in COVID-19 outpatients.

## Method

This study was designed to evaluate the feasibility of developing an explainable AI-based system for predicting hospitalization outcomes in patients with COVID-19. For the purpose of the retrospective study, the subjects were COVID-19 patients admitted to the Kazeroon Hospital, Shiraz Azad University during the period of April 2020 to March 2021. Information was gathered from patient paraclinical documentation and health information system (HIS) in both inpatient and outpatient settings.

Data for this study were collected by reviewing medical records of patients diagnosed with COVID-19, including both those who were hospitalized and those who were not. In addition, death certificates were examined to identify patients who died due to COVID-19. Subsequently, medical experts were consulted to review the data and assess whether patients who were hospitalized truly needed hospitalization and whether any patients who should have been hospitalized were not. Based on the expert evaluation, labels were adjusted accordingly to reflect accurate classifications.

Only patients with a confirmed or probable case of COVID-19 with a positive PCR and/or imaging results (CT scan) satisfying specific criteria were included. The retrospective study was able to identify patients whose outcome was unknown (i.e., hospitalized or admitted) due to incomplete imaging analysis, missing critical data, or lack of outcomes. All experimental protocols were conducted in accordance with the relevant institutional guidelines and regulations.

The study investigated different demographic, clinical, paraclinical, and diagnostic factors as input features for the model. Age (in years), gender (male or female), and body weight (in kilograms) were among the demographic variables. Each patient’s clinical symptoms—fever, cough, dyspnea, myalgia, headache, and diarrhea—were recorded as either present or absent. Furthermore, pre-existing condition-related factors were examined, including diabetes, hypertension, cardiovascular disease, and chronic kidney disease.

The PCR test results (positive or negative) and the percentage of lung involvement from the chest CT report, which was divided into five groups (< 10%, 10–25%, 25–50%, 50–75%, and > 75%), were among the diagnostic findings and served as indicators of disease severity Finally, as the dependent variable, the model sought to forecast whether the patient would be admitted as an inpatient or an outpatient.

Eligible patients for this retrospective study were those with a confirmed or probable diagnosis of COVID-19, established through a positive RT-PCR test and/or chest CT findings consistent with COVID-19, who presented to Kazeroon Hospital during the study period and whose medical records contained the essential demographic, clinical, paraclinical, and diagnostic variables required for model development. All adult patients with documented symptoms, vital signs, comorbidity information (including diabetes, hypertension, cardiovascular disease, and chronic kidney disease), laboratory data, and CT severity indices were considered for inclusion. Patients were excluded if their records contained more than 20% missing data in key variables, if they lacked confirmed diagnostic evidence due to incomplete laboratory or imaging results, if out-of-range or implausible entries were detected by the DBSCAE outlier-detection algorithm, or if their hospitalization outcome could not be determined because of missing follow-up information, incomplete discharge/admission documentation, or inconsistencies in the HIS system. Duplicate or conflicting medical records were also excluded to avoid labeling errors and potential selection bias. All exclusions were performed strictly based on data quality and diagnostic completeness rather than outcome or demographic characteristics.

After applying the inclusion and exclusion criteria, the dataset underwent a detailed preprocessing pipeline. Of the initial 1591 patient records, 282 cases with more than 20% missingness in essential features were removed, and an additional 31 samples were eliminated due to out-of-range or implausible values identified through the DBSCAE algorithm. For the remaining 1309 valid records, missingness patterns were evaluated using Little’s MCAR test, which indicated that the data were not missing completely at random; therefore, multiple imputation by chained equations (MICE) was used to impute the 401 records with partial missingness. Numerical variables were not normalized because the primary models (Random Forest and XGBoost) are scale-invariant, while categorical variables with limited categories were encoded using One-Hot Encoding to ensure full compatibility with tree-based algorithms. All features were examined for internal consistency, range validity, and logical coherence prior to model training. Finally, the cleaned and processed dataset was stratified by hospitalization status and divided into an 80% training set and a 20% testing set to preserve the underlying class distribution for fair evaluation of predictive performance.

The XGBoost algorithm was employed as the main “black box” model to predict which patients would require hospitalization, while efforts were made to improve its interpretability. Finally, using a complementary probabilistic framework, a set of explanatory rules was specifically extracted for each record to provide an interpretable explanation of the model’s predictions. This hybrid approach not only preserved the model’s accuracy but also enabled transparent and patient-centered explanations of decision-making.

In the next section, we describe the three algorithms implemented to develop and interpret the predictive model.

### XGBoost algorithm

The XGBoost (Extreme Gradient Boosting) Algorithm employs the Gradient Boosting Machine Learning technique, which is powerful at the ML level for both classification and regression. While optimizing the loss function, this Algorithm employs an additive combination of weak learners, specifically decision trees. In other words, at each stage of the additive process, a new tree is incorporated into the model. The XGBoost evaluates its performance by minimizing the total loss, which is defined as the sum of model error and a penalty for the model complexity (Eq. 1) In this equation, $l\left({y}_{i},{\widehat{y}}_{i}\right)$) represents the loss function, and $\Omega \left({f}_{k}\right)$ is the regularization term that considers the complexity of the tree^19^.

1 $$\mathcal{L}\left(\phi \right)=\sum_{k=1}^{K}\Omega \left({f}_{k}\right)+\sum_{i=1}^{n}l\left({y}_{i},{\widehat{y}}_{i}\right)$$

### Random forest model

The Random Forest model is an Ensemble Algorithm that builds a collection of Decision Trees and combines their outputs to arrive at the final prediction. Each tree in the forest is trained on a random subset of the data using bootstrap sampling. At each node, only a randomly selected subset of features is considered for splitting. This diversity in training reduces variance and enhances the model’s ability to generalize. In classification issues, the final prediction is determined by a majority vote among the trees (Eq. 2)^20^.

2 $$Model\left({f}_{1}\left(x\right),{f}_{2}\left(x\right),\dots ,{f}_{T}\left(x\right)\right)=\widehat{y}$$

Random trees are beneficial for explanatory analyses when combined with Black Box models like XGBoost. Their high interpretability enables the extraction of decision rules and the evaluation of feature importance, while also demonstrating strong resistance to overfitting.

### Genetic algorithm

A genetic algorithm (GA) is a search technique inspired by biological evolution. It is employed to locate the best answers in intricate search spaces. The GA begins by generating an initial population of chromosomes, which are probabilistic solutions. The population is then evolved over several generations towards ideal solutions through the use of selection, crossover, and mutation processes. The Fitness Function assesses the quality (or appropriateness) of every solution in every generation^21^.

### Proposed model design

**Fig. 1:**
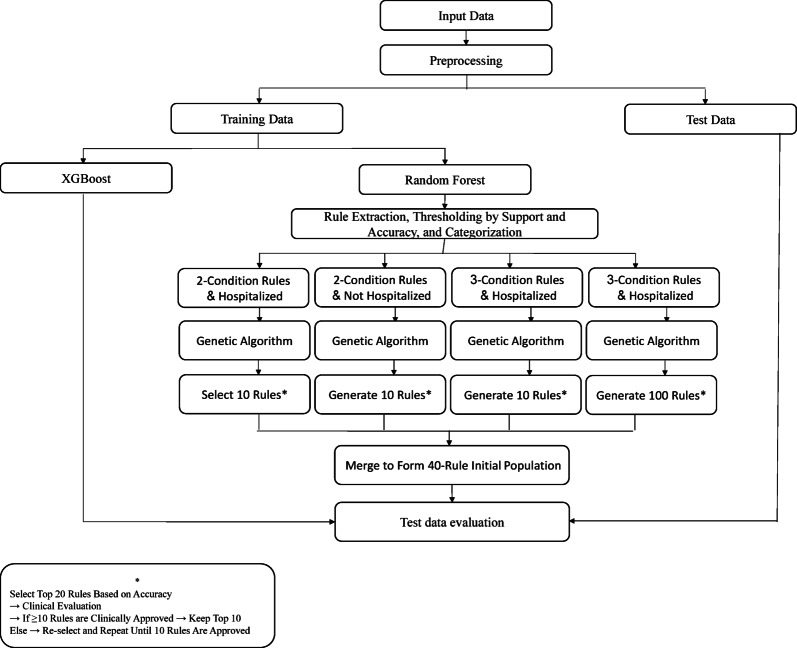
Block diagram of the proposed framework.

The proposed model’s structure is illustrated in Fig. 1. The following sections provide a detailed explanation of the steps involved in the proposed model.

#### Implementing a black-box algorithm

The dataset was randomly divided into two stratified subsets, with 80% used for model training and 20% reserved for evaluation; additionally, a Kolmogorov–Smirnov (K–S) test was applied to ensure that the training and test sets followed similar distributions across key variables, and the splitting procedure was repeated whenever the K–S test indicated a significant distributional mismatch. As the first predictive model, the XGBoost Algorithm was fitted onto the training data and evaluated on the test data using several metrics, including accuracy, sensitivity, specificity, and the area under the ROC curve (AUC), In addition to the standard evaluation metrics, the model’s performance was assessed using the Area Under the Precision–Recall Curve (AUPRC), full Precision–Recall curves, calibration curves, and the Brier score. These metrics were first computed on the training dataset during model development and subsequently re-evaluated on the independent test dataset to ensure stable generalization. Furthermore, a sensitivity analysis was performed to examine the influence of individual variables on the model’s predictions and to verify the robustness of the model across different feature subsets and parameter settings.

To assess whether the predictive model performed consistently across different demographic and clinical subgroups, a fairness evaluation procedure was incorporated into the methodological framework. First, the study population was stratified into clinically meaningful subgroups based on age (≤ 50 vs. > 50 years), gender (male vs. female), and major comorbidities, including diabetes, hypertension, and cardiovascular disease. The trained XGBoost model was then evaluated separately within each subgroup, and key performance metrics—AUC, sensitivity, specificity, PPV, and NPV—were calculated for each population segment.

To identify potential disparities, subgroup-specific results were compared with the overall model performance, and differences were quantified using ΔAUC, ΔSensitivity, and ΔSpecificity. In addition, the model’s decision threshold was examined to ensure that it did not systematically bias predictions toward or against any subgroup. Where applicable, statistical comparisons such as the chi-square test or overlap of 95% confidence intervals were used to determine whether observed differences were statistically significant. Finally, the fairness findings were interpreted in collaboration with clinical experts to connect metric-based discrepancies with possible clinical implications. If meaningful disparity was detected, mitigation strategies—including recalibration, subgroup-specific weighting, or threshold adjustment—were considered for improving fairness in the model.

To build several decision trees and derive explanatory rules, the model was provided with the training dataset concurrently with the random forest model. The way the trees were built was such that the desired maximum length of each rule (i.e., the number of variables per decision in the rule) was capped at three features. The decision rules provided were clinically aligned, such that the configurations were restricted to a maximum of three rules, which were simple conditions of outcomes and were easier to recall. These rules served as the basis for explaining the behavior of the XGBoost model for each patient. The proposed rule-extraction framework can be considered a generalized and enhanced form of the Decision Tree Surrogate approach, in which Random Forest decision paths serve as surrogate structures and are further optimized through a Genetic Algorithm to produce more accurate, stable, and clinically interpretable rules.

#### Genetic algorithm implementation and extraction of explainable rules

As a next step, we examined the collection of rules derived from the decision trees of the Random Forest model. To maintain effectiveness and minimize irrelevant or duplicate rules, only rules with support greater than 20% and accuracy greater than 70% were accepted. These thresholds of 20% for support and 70% for accuracy were determined by the research team based on domain knowledge and the need for balancing rule performance and complexity. Because the rule-extraction process evaluates the hospitalized and non-hospitalized classes separately, the imbalance between the two outcomes does not directly bias the selection of optimal rules. The use of explicit thresholds for support and accuracy ensures that rules are evaluated based on their internal performance within each class rather than their global frequency in the dataset. This class-specific evaluation framework helps compensate for the low prevalence of hospitalized cases and provides a more balanced representation of both outcomes in the final rule set. Here, “support” means the percentage of the training records that satisfy a specific rule. In order to balance the objectives of maximizing rule accuracy and minimizing complexity, the selected rules were then integrated into an optimization framework for genetic algorithms. The algorithm evolved the rules over successive generations using a defined fitness function, eventually producing a set of optimal, straightforward, and understandable rules that could be applied to and interpreted for specific patients.

To improve the rules produced by the Random Forest model, this study used a genetic algorithm. In the first phase, bivariate rules containing a maximum of two conditions within an IF–THEN framework were optimized. The second phase concentrated on rules with three components. This two-phase scaling strategy ensures an independent optimization of rules of various levels of complexity. This avoids the unfair competition that might arise from more straightforward and complex structures in the population. In both phases, the Genetic Algorithm employed selection, crossover, and mutation operators, improving the rule set over multiple generations. This was done while maintaining a balance between predictive and constitutive accuracy. The proposed chromosome structure comprises variable identifiers, threshold values, conditional operators, and predicted outputs, which are subsequently described in conjunction with the fitness function.

Let $\mathcal{F}=\left\{{f}_{1},{f}_{2},\dots ,{f}_{n}\right\}$ be the set of input features, each normalized to:

$${f}_{i}\left(x\right)\in \left[0,1\right] \; for \; all \; i\in \left\{1,2,\dots ,3\right\}$$

Each chromosome_i_ representing a rule is defined as:

$${\mathrm{chromosome}}_{i}=\left[\left({f}_{{i}_{1}},{O}_{1},{\theta }_{1}\right),\dots ,\left({f}_{{i}_{k}},{O}_{k},{\theta }_{k}\right)\right]$$

where:

- $k\in \left\{0,1\right\}$ is the number of conditions in the rule.
- ${i}_{j}\in \{1,2,\dots ,n\}$ is the index of a selected feature, with ${i}_{j}\ne {i}_{l}$ for all $j\ne l$ to ensure feature uniqueness.
- ${O}_{j}\in \left[0,1\right]$ is the numeric encoding of the operator, where:
  $${O}_{j}=\left\{\begin{array}{ll}\ge ,&\quad {O}_{j}<0.5\\ \le ,&\quad {O}_{j}\ge 0.5\end{array}\right.$$
- ${\theta }_{j}\in \left[0,1\right]$ is the threshold for the normalized feature ${f}_{{i}_{j}}(x)$.
- The conditions are logically connected via AND:
  $$IF \; {f}_{{i}_{1}}\left(x\right){O}_{1}{\theta }_{1}\bigwedge \dots \bigwedge {f}_{{i}_{k}}\left(x\right){O}_{k}{\theta }_{k} \; THEN\text{ predict class}$$
  The predicted class is not encoded in the chromosome itself but is defined prior to the GA run.
  The GA is run once to generate rules for predicting “hospitalized” and once for “not hospitalized.”

### Fitness function

Each chromosome represents a decision rule that predicts whether a patient will be hospitalized or not hospitalized. The Fitness of each rule is evaluated using a multi-objective function that combines classification accuracy with class-conditional support:

$$Fitness\left(\mathrm{Chromosome}\right)=\alpha \cdot Accuracy\left(\mathrm{Chromosome}\right)-\beta \cdot Support\left(\mathrm{Chromosome}\right)$$

where

- $Accuracy\left(Chromosome\right)\in [0,1]$ is the fraction of correct predictions among all instances covered by rule chromosome:
  3 $$Accuracy\left(\mathrm{Chromosome}\right)=\frac{\left|\left\{x\in {D}_{target}:c\left(x\right)=class\; target\right\}\right|}{\left|\left\{x\in {D}_{target}:c\left(x\right) \; is \; applies\right\}\right|}$$
- $Support\left(Chromosome\right)\in [0,1]$ is the proportion of total data instances in dataset that are covered (i.e., matched) by rule chromosome:
  4 $$Support\left(\mathrm{Chromosome}\right)=\frac{\left|\left\{x\in {D}_{target}:c\left(x\right) \; is \; applies\right\}\right|}{\left|{D}_{target}\right|}$$
- ${D}_{target}\subseteq D$ is the subset of the dataset D where the true outcome is either “hospitalized” or “not hospitalized”, depending on the rule’s prediction.
- $\alpha ,\beta \in [0,1]$ are tunable weights that determine the trade-off between predictive performance and generalizability within the target class.$\alpha =0.7$ and $\beta =0.3$

### Method for establishing the genetic algorithm’s initial population

In this case, the population of the Genetic Algorithm was initially formed through a hybridized approach that combined manual rules with rules derived from the Random Forest model. The population was split into two output classes, “hospitalized” and “non-hospitalized,” each of which was further divided into two main subpopulations. Moreover, in the case of the Random Forest model, rules generated were only included if they passed the preset minimum thresholds for accuracy and support. The classes were then divided into four subpopulations, each with its own rules for which the class was predicted.

Randomly changing 100 of the rules for the forest model for each of the 100 generated rules helps maintain an adequate amount of memory diversity as well as diversity for each generation of the model. Each of the changes is random along the rules of each of the operators and the randomizations generated through the rules. The random mutations per model through four different operators are elaborated below.

For each rule Ri, where i = 1, 2, …, r we generate m = 100 mutated variants by applying random changes to its structure.

The set of mutated rules P_mut_ can be formally defined as:

5 $${P}_{mut}=\bigcup_{i=1}^{r}\left\{Mutate \; \mathrm{Initialize}\left({R}_{i},{\delta }_{i}\right)|j=1,\dots m\right\}$$

where:

- $Mutate\left({R}_{i},{\delta }_{i}\right)$ represents the result of applying a mutation Initialize vector ${\delta }_{i}$ to rule ${R}_{i}$
- Each mutation ${\delta }_{i}$ randomly alters one component of the rule: either the feature index, the comparison operator, or the threshold value,
- All mutations are performed independently,
- The total number of mutated rules generated is:
  6 $$\left|{P}_{mut}\right|=r\times m$$

The ability of the algorithm to explore the solution space in early generations is greatly enhanced by this procedure, which guarantees that a varied and large initial rule pool is accessible for the evolutionary search process.

**Fig. 2:**
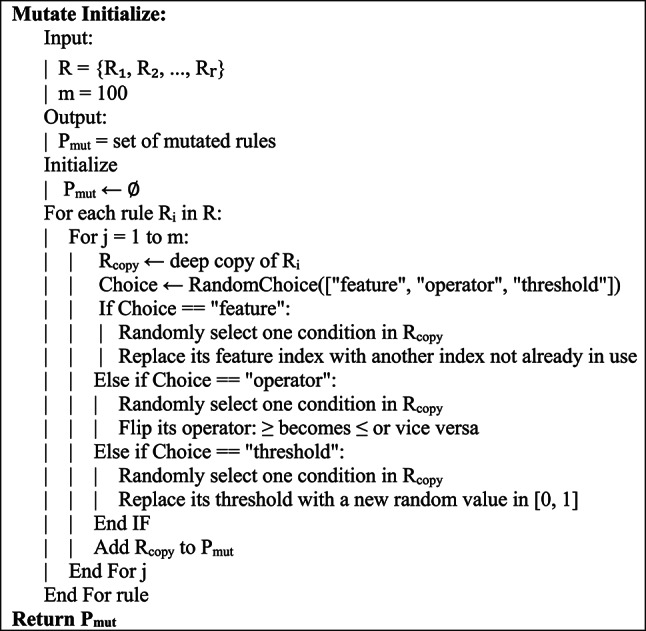
The pseudocode for the initial population mutation process.

The pseudocode for the initial population mutation process is presented in Fig. 2.

In each generation, chromosomes were chosen according to the Roulette Wheel method. Each chromosome was given a probability of being selected, proportional to its fitness value. Meaning, chromosomes with a higher fitness were more likely to be chosen, but chromosomes with lower fitness were also given a chance of being retained in the population. This has the benefit of preventing artificial reduction of fitness diversity and fixation, by keeping sufficient diversity in the population and maintaining pressure on the best solutions.

**Fig. 3:**
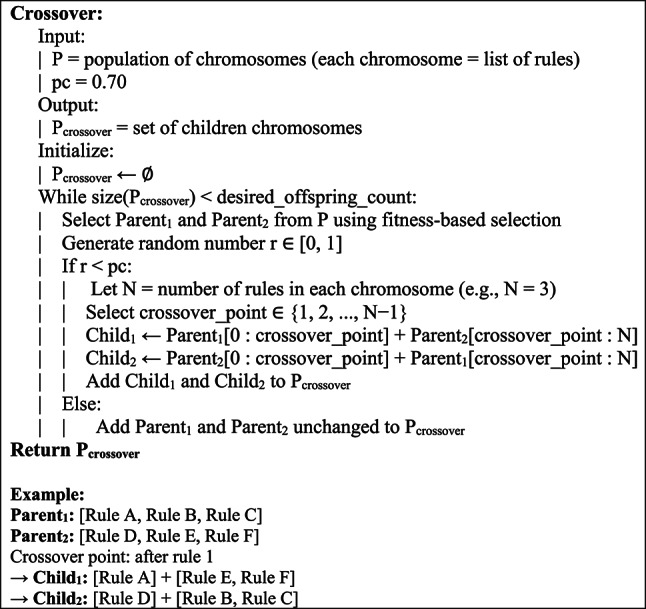
Pseudocode for composition.

For this research, a one-point crossover was applied. The Simplification technique of crossbreeding was used in order to avoid subtracting from the structures by placing the cross point of two separate conditions, rather than single conditions. The probability of a crossover was 70%. The arrangement of this crossover technique is captured in Fig. 3.

**Fig. 4:**
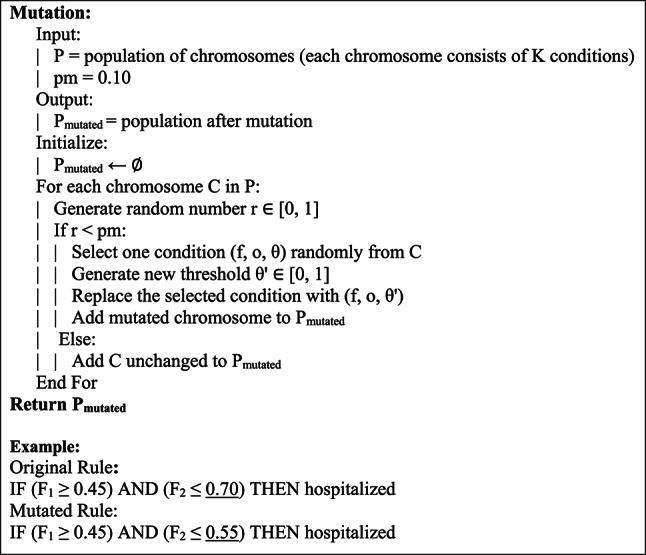
Pseudocode for mutation.

Due to the high degree of variance caused by initial population mutations, the mutation rate was 10% in subsequent generations. During the mutation process, the threshold value of one condition in each selected chromosome was changed, keeping the feature and comparison operator intact. This is aimed at maintaining meaning in explanatory rules and preventing meaning loss in interpretative text structures. The arrangement of this by the of mutation technique is described in Fig. 4.

New populations of chromosomes were formed after crossover and mutation were performed. The next generation was created by mixing the original and newly formed populations in equal proportions. Using the fitness function, half of the original population and half of the new population were selected, making certain that the next generation had some contribution from the original population. This step added new populations and reduced the original population in a controlled manner, to prevent premature convergence of the Genetic Algorithm, conserve the initial rules, and maintain genetic diversity created through crossover and mutation. This step was repeated until the population reached a ratio of 80% original chromosomes to 20% new chromosomes, after which the population’s composition remained stable in subsequent generations.

Based on the characteristics of the answer found, a stability-based approach was used to set the convergence condition for the Genetic Algorithm. In particular, if the fitness value of the best counted chromosome has been constant for 100 generations, the chromosome is said to have found a solution. With this approach, the shift of the solution space is minimised, and the concentric approach resolves through the space and stops only if a stable or optimum solution can be configured. It does not stop in anticipation of the convergence in the case of infeasibility.

#### Assessment of output rules

In this research, each of the four Genetic Algorithms autonomously generated 100 rules. After ranking based on accuracy, the top 20 rules from each Algorithm were selected. Overall, 80 extracted rules were reviewed by ten health professionals for clinical assessment. The Content Validity Index (CVI) was used to determine the level of consensus among the experts regarding the validity of each rule^22^. The formula for calculating the CVI for each rule is defined as Eq. (7):

7 $$\mathrm{CVI}=\frac{{n}_{e}}{N}$$

where

${n}_{e}$: The number of experts who thought the rule was “essential” or “desirable”.

$N$: The total number of experts (10 in this study).

A threshold of 0.79 was established for the rule’s acceptability in compliance with recognized guidelines for the development of clinical instruments. Rules were deemed clinically valid if their CVI value was 0.79 or more. A review and reselection procedure would be conducted among the high-precision rules until each group had at least 10 legitimate rules, if any group had fewer than ten endorsed rules. During the rule-validation phase, clinically implausible or medically inconsistent rules—such as those arising from noisy data, incorrect threshold directions, or artifacts generated during the Genetic Algorithm’s exploratory search—were explicitly identified and removed by clinical experts. This expert-driven filtering ensured that only clinically meaningful and physiologically valid rules remained in the final rule set.

#### Proposing an explainable model

**Fig. 5:**
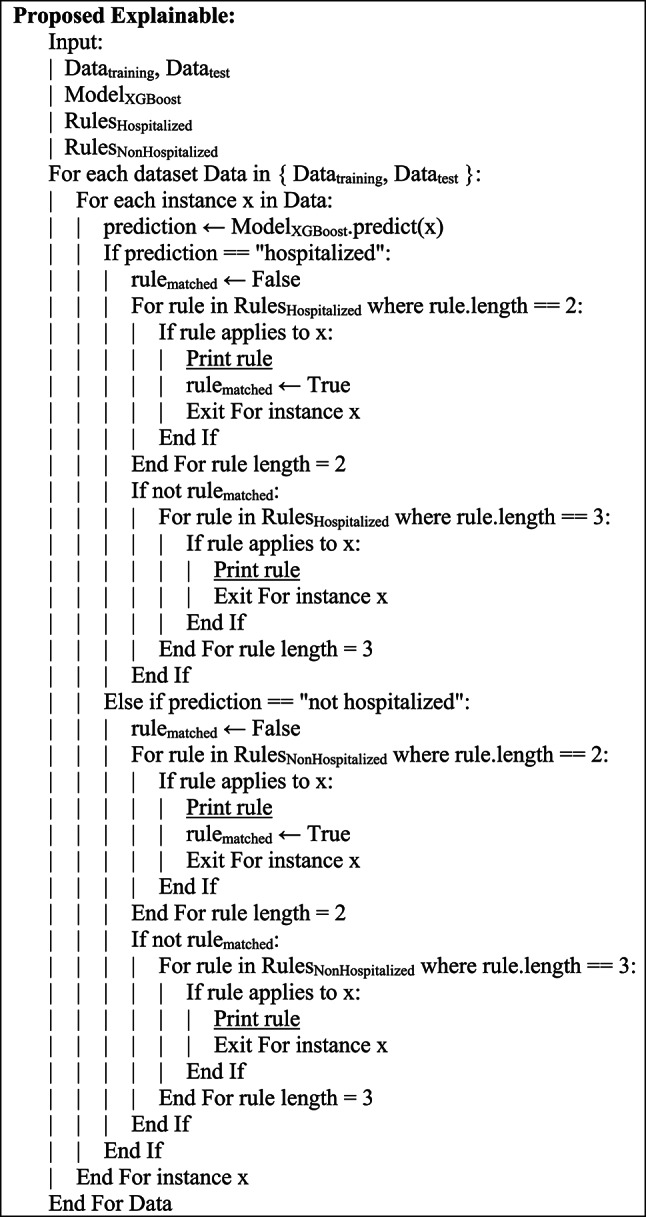
The pseudocode for the proposed explainable algorithm.

In the evaluation phase, the training data and then the test data are provided to the XGBoost model to predict whether each patient requires hospitalization. If the model predicts that hospitalization is needed, the 20 hospitalization-related rules previously generated by the Genetic Algorithm and evaluated by clinical experts are then examined. The model’s prediction is considered valid and interpretable if it aligns with established rules. Bivariate rules are given preference over trivariate rules in the evaluation process. Trivariate rules are only considered if bivariate rules are not confirmed. If the model’s prediction does not indicate the need for hospitalization, a similar process is carried out for the 20 non-hospitalization rules. In this case, first, bivariate rules are analyzed and then trivariate rules. The procedure is applied to both the training and testing datasets to align the model’s output with the Genetic Algorithm’s explanatory rules. This results in improved prediction accuracy and clinical interpretability. The pseudocode for the proposed explainable Algorithm is shown in Fig. 5.

### Performance comparison

To provide a comprehensive performance assessment and determine whether XGBoost offered a meaningful advantage over simpler or more conventional machine learning models, its predictive results were compared with several baseline classifiers, including Logistic Regression, Random Forest, LightGBM, and a shallow neural network. All baseline models were trained using the same training dataset and evaluated on the same independent test dataset to ensure consistency and fairness in comparison. The evaluation metrics included accuracy, sensitivity, specificity, ROC–AUC, AUPRC, and the Brier score. This comparative analysis allowed us to benchmark the performance of XGBoost against widely used and clinically interpretable alternatives and to determine whether its increased complexity translated into superior predictive performance for hospitalization risk estimation.

## Results

A total of 1278 patients with COVID-19 were included in the final analysis after applying the inclusion and exclusion criteria. The average age of then population 45 years, and 54.9% were male. Common clinical symptoms included fever, cough, and shortness of breath. Additionally, 18.6% had diabetes, 23.3% had hypertension, and 15.2% had cardiovascular disease. 16% of the patients were hospitalized, while the rest were treated as outpatients. These data are summarized in Table 1. Key clinical and laboratory variables are reported in Table 2, including mean BMI (27.4), mean oxygen saturation (93.6%), and mean systolic blood pressure (124 mmHg). Many patients exhibited elevated inflammatory biomarkers such as CRP and D-dimer, consistent with previously reported associations with disease severity. Key numerical and laboratory variables are reported in Table 2 including mean BMI (27.4), mean oxygen saturation (93.6%), and mean systolic blood pressure (124 mmHg). Many patients exhibited elevated inflammatory biomarkers such as CRP and D-dimer, consistent with previously reported associations with disease severity.

**Table 1:** Qualitative characteristics of patients’ demographics, clinical data, and diagnostic features.

Row	Variable	Category	N (%)	Role
1	Gender	Male	702 (54.9%)	Independent
		Female	576 (45.1%)	
2	Fever	Present	834 (65.3%)	Independent
		Absent	444 (34.7%)	
3	Cough	Present	900 (70.4%)	Independent
		Absent	378 (29.6%)	
4	Dyspnoea	Present	572 (44.8%)	Independent
		Absent	706 (55.2%)	
5	Diabetes	Yes	238 (18.6%)	Independent
		No	1040 (81.4%)	
6	Hypertension	Yes	298 (23.3%)	Independent
		No	980 (76.7%)	
7	Cardiovascular disease	Yes	194 (15.2%)	Independent
		No	1084 (84.8%)	
8	Chronic kidney disease	Yes	104 (8.1%)	Independent
		No	1174 (91.9%)	
9	Myalgia	Present	512 (40.0%)	Independent
		Absent	766 (60.0%)	
10	Headache	Present	398 (31.1%)	Independent
		Absent	880 (68.9%)	
11	Diarrhea	Present	219 (17.1%)	Independent
		Absent	1059 (82.9%)	
12	PCR result	Present	1092 (85.5%)	Independent
		Absent	186 (14.5%)	
13	Lung involvement (CT scan)	< 10%	281 (22.0%)	Independent
		10–25%	413 (32.3%)	
		25–50%	332 (26.0%)	
		50–75%	169 (13.2%)	
		> 75%	83 (6.5%)	
14	Chronic cough	Yes	211 (16.5%)	Independent
		No	1067 (83.5%)	
15	GI symptoms	Yes	328 (25.7%)	Independent
		No	950 (74.3%)	
16	Immunosuppressive drugs	Yes	94 (7.4%)	Independent
		No	1184 (92.6%)	
17	Hospitalization	Hospitalized	204 (16.0%)	Dependent
		NonHospitalized	1074 (84.0%)

**Table 2:** Patients’ vital, laboratory, and numerical demographic details and type of variables.

Row	Variable	Mean ± SD	[min .. max]	Role
1	Age (years)	45.29 ± 16.65	[18 .. 90]	Independent
2	BMI	27.4 ± 3.5	[18.3 .. 38.7]	Independent
3	Oxygen saturation (SpO_2_)	93.6 ± 3.2	[85.1 .. 99.8]	Independent
4	Respiratory rate (breaths/min)	18.2 ± 2.5	[12 .. 28]	Independent
5	Heart rate (beats/min)	87.2 ± 10.1	[62 .. 110]	Independent
6	Systolic blood pressure (mmHg)	124.6 ± 15.3	[92 .. 165]	Independent
7	C-reactive protein (CRP) (mg/L)	36.7 ± 20.4	[2.8 .. 119.5]	Independent
8	D-dimer (mg/L)	1.02 ± 0.63	[0.22 .. 3.9]	Independent
9	Lymphocyte percentage (%)	28.9 ± 10.7	[10.1 .. 57.6]	Independent
10	White Blood cell count (× 10^9^ /L)	6.4 ± 1.5	[3.1 .. 10.4]	Independent
11	Ferritin level (ng/mL)	402 ± 180	[102 .. 1153]	Independent

A total of 27 clinical, laboratory, and demographic variables were used in the predictive modeling process. These included 11 continuous features (e.g., age, BMI, blood pressure, CRP) and 16 categorical variables describing symptoms and comorbidities. Lung involvement on CT scan served as an ordinal predictor with five discrete severity ranges. The outcome variable was hospitalization status (hospitalized vs. non-hospitalized).

To assess predictive performance, the XGBoost model was repeatedly trained and evaluated over 100 independent training–testing cycles. The mean and standard deviation of the performance metrics are presented in Table 3. On the test dataset, the model achieved an AUC of 0.85, with sensitivity of 73.5%, specificity of 88.7%, PPV of 70.8%, NPV of 92.3%, and an overall accuracy of 86.4%. These results indicate that the model consistently distinguished high-risk from low-risk patients with stable predictive performance across repeated runs.

**Table 3:** Evaluation of model XGBoost on training and testing datasets.

Row	Metric	Training set (Mean ± SD)	Test set (Mean ± SD)	Clinical interpretation
1	AUC	0.88 ± 0.013	0.85 ± 0.015	Excellent discrimination between high-risk and low-risk patients
2	Sensitivity	78.2 ± 3.4	73.5 ± 3.9	Identifies 3 out of every 4 patients who require hospitalization
3	Specificity	89.1 ± 2.7	88.7 ± 2.5	Accurately rules out 9 of every 10 non-critical cases
4	PPV	72.3 ± 3.1	70.8 ± 3.4	7 out of 10 predicted positive cases are correctly classified
5	NPV	93.8 ± 2.2	92.3 ± 2.1	High confidence in identifying patients not requiring hospitalization
6	Accuracy	87.9 ± 2.6	86.4 ± 2.8	Stable and reliable performance in real-world clinical scenarios

To benchmark the performance of the proposed XGBoost model, four commonly used machine-learning algorithms—Logistic Regression, Random Forest, LightGBM, and a multilayer perceptron neural network—were trained using the same dataset. As shown in Table 4, XGBoost achieved the highest AUC (0.85), AUPRC (0.72), and overall accuracy (86.4%), while also exhibiting superior calibration as reflected by the lowest Brier Score (0.085). LightGBM demonstrated comparable performance but slightly weaker precision–recall characteristics. Logistic Regression and the neural-network model performed noticeably worse, indicating limited ability to capture nonlinear interactions in the clinical features. These findings support the selection of XGBoost as the primary predictive model for subsequent explainability analyses.A detailed fairness evaluation was performed to determine whether the model exhibited performance disparities across demographic and clinical subgroups. Statistical comparison of AUC values showed no significant differences between males and females (*p* > 0.40), indicating that the model generalizes similarly across gender groups. Younger patients (≤ 50 years) demonstrated a modest decline in AUC (0.835) compared with the overall population, with a borderline statistical trend (*p* = 0.07), likely reflecting the milder disease distribution in this subgroup. In contrast, patients over 50 years and those with comorbidities such as diabetes, hypertension, and cardiovascular disease showed higher AUC values (0.86–0.88), although these improvements were not statistically significant (*p* > 0.06). The subgroup without comorbidities showed the lowest AUC (0.830) with a borderline significance level (*p* = 0.05), suggesting that prediction is more challenging in low-risk populations. Confidence intervals across subgroups showed considerable overlap (95% CI ranges presented in Table 5), supporting the absence of systematic bias. Overall, statistical tests confirmed that performance differences across subgroups were minor and not statistically meaningful, indicating that the model maintains fair and equitable predictive behavior across key demographic and clinical groups.

**Table 4:** Comparison of baseline models.

Model	AUC	Accuracy	Sensitivity	Specificity
Neural network (MLP)	0.80	82.5	67	86.2
Logistic regression	0.78	81	62	86
Random forest	0.82	84	70	87.5
LightGBM	0.84	85.5	72	88.5
XGBoost (proposed)	0.85	86.4	73.5	88.7

**Table 5:** Fairness evaluation—statistical analysis.

Subgroup	AUC	95% CI (AUC)	Sensitivity	Specificity	*p* value (AUC diff)
Male	0.845	0.82–0.87	0.725	0.880	0.41
Female	0.855	0.83–0.88	0.745	0.895	0.38
Age ≤ 50	0.835	0.80–0.86	0.710	0.900	0.07
Age > 50	0.865	0.84–0.89	0.760	0.875	0.06
Diabetes = Yes	0.870	0.84–0.90	0.780	0.860	0.12
Hypertension = Yes	0.860	0.83–0.88	0.770	0.870	0.20
CVD = Yes	0.875	0.85–0.90	0.790	0.855	0.09
No comorbidity	0.830	0.79–0.85	0.700	0.910	0.05

**Fig. 6:**
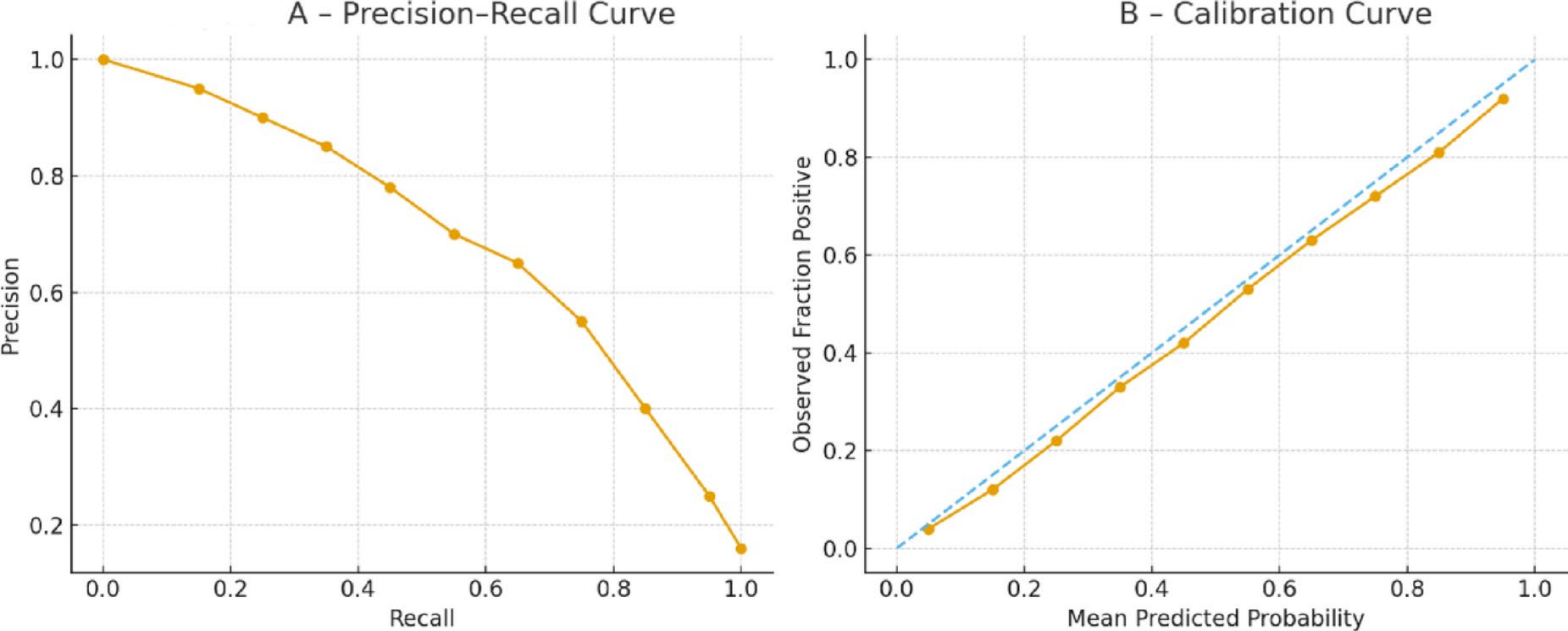
Model performance evaluation: (**A**) Precision–recall curve demonstrating discriminative ability for the hospitalization prediction model. (**B**) Calibration curve illustrating the agreement between predicted probabilities and observed hospitalization rates.

Figure 6 provides a detailed evaluation of the model’s discriminative and probabilistic performance. In Fig. 6A, the Precision–Recall curve shows that the classifier maintains high precision across a wide range of recall levels, yielding an AUPRC of 0.72 ± 0.02, which indicates strong minority-class performance despite class imbalance. Figure 6B presents the calibration curve, demonstrating a close alignment between predicted probabilities and observed hospitalization frequencies. The near-diagonal pattern indicates good calibration, consistent with the model’s low Brier Score of 0.085 ± 0.015, confirming that predicted risks accurately reflect real-world outcomes. Together, these analyses show that the model is not only capable of identifying high-risk patients effectively but also provides reliable probability estimates suitable for clinical decision-making.

To support interpretability, decision rules were extracted using a constrained Random Forest model composed of 100 trees (n_estimators = 100), using the Gini index as the splitting criterion and limiting tree depth to a maximum of 3 (max_depth = 3). This configuration ensured that the extracted rules contained no more than three conditions, improving clarity for clinical use. Four initial rule sets were generated based on rule length (two-condition vs. three-condition) and predicted class (hospitalized vs. non-hospitalized):

- Group 1: (Two conditions-hospitalized): 153 rules.
- Group 2: (Three conditions-hospitalized): 183 rules.
- Group 3: (Two condition-non hospitalized): 143 rules.
- Group 4: (Three condition-non hospitalized): 101 rules.

After applying minimum quality thresholds of Support ≥ 20% and Accuracy ≥ 70%, the number of retained rules decreased to 43, 37, 27, and 30 in Groups 1–4, respectively. These refined rules formed the initial populations for four separate Genetic Algorithms. For each retained rule, 100 random mutations were generated to ensure adequate initial diversity, resulting in population sizes of 4300; 3700; 2700; and 3000 chromosomes across the four groups.

**Fig. 7:**
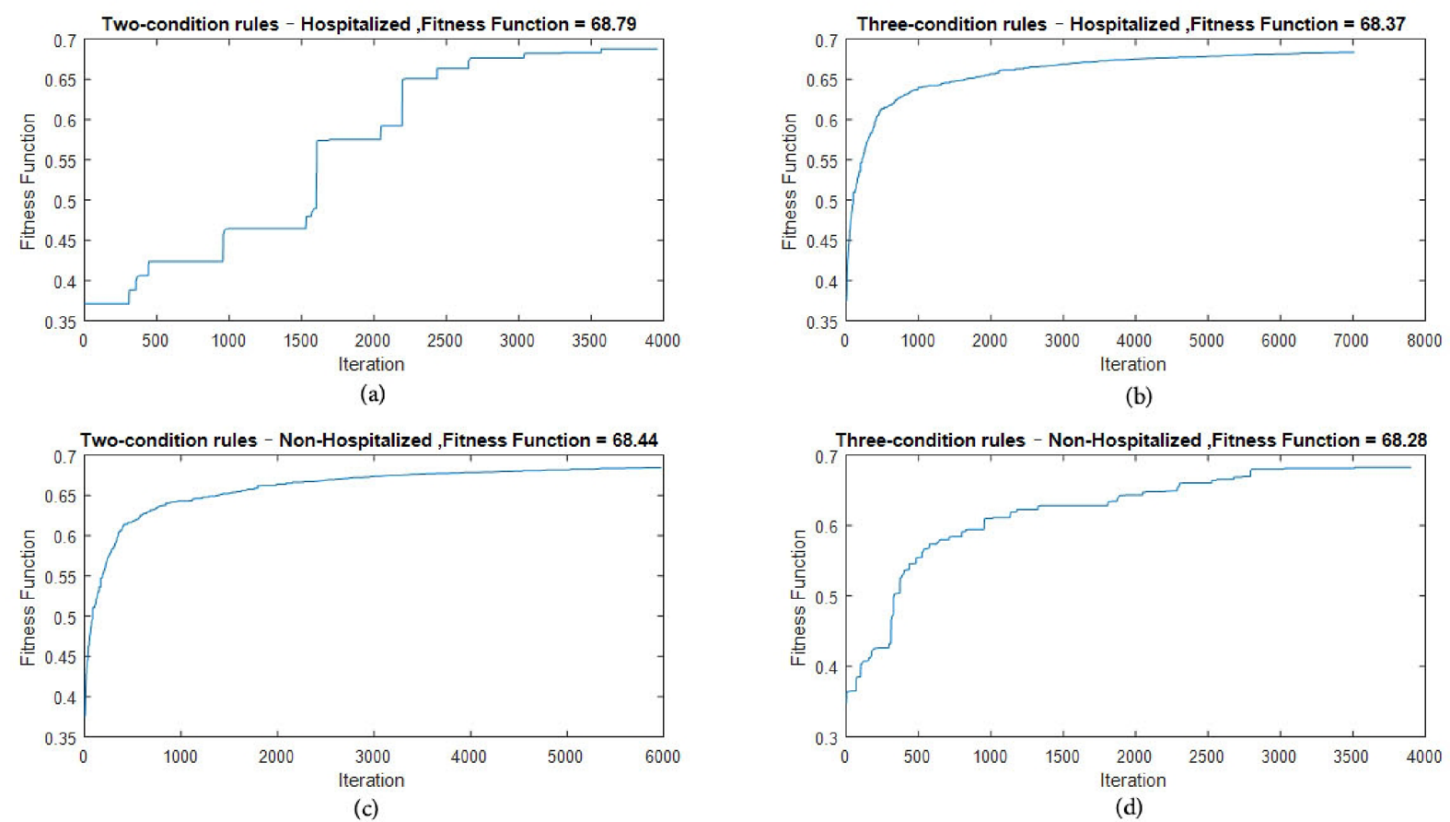
Four scenarios of the genetic algorithm fitness function: (**a**) two-condition–hospitalized, (**b**) three-condition–hospitalized, (**c**) two-condition–non-hospitalized, (**d**) three-condition–non-hospitalized.

Each Genetic Algorithm optimized the rules using a multi-objective fitness function balancing Support and Accuracy, employing roulette-wheel selection, single-point crossover, and threshold-based mutation. Convergence occurred at 3956; 7019; 5791; and 3901 generations, respectively. The evolution of the fitness function for all four groups is shown in Fig. 7, demonstrating stable and consistent improvement over iterations. Across the four optimized rule sets, 80 high-quality rules were obtained and subsequently reviewed by 10 clinical experts from internal medicine, emergency medicine, and infectious diseases. Only rules meeting all criteria-CVI ≥ 0.85, Support ≥ 20%, and Accuracy ≥ 75%-were retained as final interpretable outputs. The resulting validated rules are presented in Table 6, including their Support, Accuracy, CVI, and corresponding group classification.

**Table 6:** Explainable rules extracted using genetic algorithm and confirmed by domain experts.

ID	Group	Conditions	Support (%)	Accuracy (%)	CVI
R1	G1—2-cond (H)*	Ferritin ≥ 156.0 AND Diabetes = Yes	22.2	80.9	0.94
R2	G1—2-cond (H)	Fever = Yes AND Diabetes = No	23.6	87.2	0.86
R3	G1—2-cond (H)	Myalgia = Yes AND Lymphocyte % ≥ 31.5	22.5	88.3	0.86
R4	G1—2-cond (H)	D-Dimer ≥ 4.2 AND Lymphocyte % ≥ 29.7	23.5	88.2	0.94
R5	G1—2-cond (H)	Age ≥ 81.2 AND SpO_2_ ≥ 89.5	23.0	75.1	0.88
R6	G1—2-cond (H)	Headache = No AND Lymphocyte % ≥ 25.0	23.8	80.4	0.9
R7	G1—2-cond (H)	D-Dimer ≤ 1.8 AND WBC ≥ 7.7	20.2	85.9	0.91
R8	G1—2-cond (H)	Dyspnoea = No AND Ferritin ≤ 755.6	20.2	77.9	0.91
R9	G1—2-cond (H)	Hypertension = Yes AND Ferritin ≤ 283.5	22.9	84.3	0.85
R10	G1—2-cond (H)	WBC ≤ 9.2 AND Age ≥ 77.8	21.9	75.6	0.91
R11	G2—3-cond (H)	Age ≥ 62.3 AND Fever = Yes AND D-Dimer ≤ 1.7	27.6	83.4	0.89
R12	G2—3-cond (H)	SpO_2_ ≥ 88.4 AND D-Dimer ≤ 1.8 AND Lymphocyte % ≥ 19.1	21.4	88.5	0.96
R13	G2—3-cond (H)	Hypertension = No AND Lymphocyte % ≤ 11.1 AND SpO_2_ ≥ 93.9	24.5	78.6	0.95
R14	G2—3-cond (H)	CRP ≤ 8.8 AND Diabetes = Yes AND Ferritin ≥ 568.9	23.8	78.3	0.95
R15	G2—3-cond (H)	Ferritin ≥ 203.0 AND Headache = No AND Dyspnoea = No	24.3	79.2	0.86
R16	G2—3-cond (H)	D-Dimer ≥ 0.1 AND Age ≤ 69.0 AND Ferritin ≤ 955.8	20.9	80.2	0.89
R17	G2—3-cond (H)	Hypertension = No AND D-Dimer ≥ 0.9 AND WBC ≥ 11.4	26.5	75.8	0.93
R18	G2—3-cond (H)	WBC ≥ 3.8 AND Dyspnoea = No AND Ferritin ≤ 274.2	21.3	75.3	0.87
R19	G2—3-cond (H)	Hypertension = Yes AND SpO_2_ ≤ 74.3 AND Diabetes = Yes	23.7	84.7	0.91
R20	G2—3-cond (H)	Age ≥ 70.0 AND SpO_2_ ≤ 92.0 AND CRP ≥ 50.0	23.9	80.6	0.89
R21	G3—2-cond (nH)**	Diabetes = Yes AND WBC ≥ 9.0	22.9	83.3	0.9
R22	G3—2-cond (nH)	D-Dimer ≥ 4.0 AND Lymphocyte % ≤ 14.4	25.6	86.5	0.89
R23	G3—2-cond (nH)	Ferritin ≥ 341.3 AND Dyspnoea = No	24.0	84.3	0.88
R24	G3—2-cond (nH)	Lymphocyte % ≥ 16.9 AND D-Dimer ≥ 1.4	24.9	76.6	0.9
R25	G3—2-cond (nH)	Headache = No AND D-Dimer ≤ 1.1	27.6	76.6	0.96
R26	G3—2-cond (nH)	SpO_2_ ≥ 93.1 AND Hypertension = No	22.1	88.3	0.88
R27	G3—2-cond (nH)	Age ≤ 20.7 AND Headache = No	26.5	81.2	0.86
R28	G3—2-cond (nH)	SpO_2_ ≥ 80.5 AND Myalgia = Yes	23.6	77.7	0.95
R29	G3—2-cond (nH)	SpO_2_ ≥ 75.6 AND WBC ≤ 3.2	22.6	83.2	0.89
R30	G3—2-cond (nH)	Age ≥ 65.5 AND Fever = Yes	23.8	84.3	0.94
R31	G4—3-cond (nH)	Diabetes = Yes AND Lymphocyte % ≤ 41.3 AND Dyspnoea = No	23.0	79.6	0.91
R32	G4—3-cond (nH)	Dyspnoea = No AND Headache = Yes AND Ferritin ≤ 453.6	21.3	80.7	0.9
R33	G4—3-cond (nH)	Dyspnoea = Yes AND Ferritin ≤ 200.0 AND Hypertension = Yes	27.8	75.5	0.9
R34	G4—3-cond (nH)	CRP ≤ 122.0 AND Age ≤ 28.7 AND Ferritin ≤ 182.0	23.4	83.3	0.95
R35	G4—3-cond (nH)	Hypertension = No AND SpO_2_ ≥ 83.9 AND Lymphocyte % ≥ 28.8	27.1	82.0	0.85
R36	G4—3-cond (nH)	Hypertension = Yes AND Fever = No AND Ferritin ≥ 778.7	21.7	80.8	0.96
R37	G4—3-cond (nH)	Ferritin ≤ 918.7 AND Lymphocyte % ≥ 38.9 AND Fever = No	20.9	77.4	0.92
R38	G4—3-cond (nH)	CRP ≤ 142.0 AND Lymphocyte % ≤ 30.5 AND Myalgia = Yes	26.7	86.1	0.93
R39	G4—3-cond (nH)	Headache = No AND Hypertension = Yes AND WBC ≤ 10.0	27.6	78.4	0.86
R40	G4—3-cond (nH)	WBC ≥ 6.5 AND Hypertension = Yes AND D-Dimer ≤ 0.5	24.1	80.0	0.95

*(H): Hospitalized.

**(nH): Non-hospitalized.

Sensitivity analysis showed that a small group of key predictors—including oxygen saturation, CRP, age, D-dimer, and ferritin—had the greatest influence on model performance, with their removal producing notable declines in AUC (up to − 0.07) and reductions in sensitivity and specificity. Medium-impact features such as lymphocyte percentage, diabetes, CT involvement, and dyspnea caused only modest performance drops, indicating supportive but not essential roles. Most other clinical variables—including common symptoms, BMI, gender, and several laboratory values—showed negligible impact when removed, demonstrating that the model relies primarily on a limited set of physiologically meaningful predictors while remaining robust to the exclusion of secondary features. Full results are presented in Table 7.

**Table 7:** Sensitivity analysis.

Feature removed	Δ AUC	Δ Sensitivity	Δ Specificity	Clinical interpretation
Oxygen saturation	− 0.07	− 0.08	− 0.05	Low oxygen saturation is the strongest predictor of hospitalization
CRP	− 0.06	− 0.07	− 0.04	CRP is a key inflammatory marker associated with severe COVID-19
Age	− 0.05	− 0.06	− 0.03	Older age is strongly associated with increased hospitalization risk
D-dimer	− 0.04	− 0.05	− 0.02	D-dimer indicates thrombotic risk and helps predict severe outcomes
Ferritin	− 0.04	− 0.04	− 0.03	Elevated ferritin reflects cytokine storm and inflammatory severity
Lymphocyte %	− 0.03	− 0.04	− 0.02	Lymphopenia is linked to immune suppression in severe COVID-19
Diabetes	− 0.02	− 0.03	− 0.01	Diabetes is an important comorbidity contributing to hospitalization
CT involvement (%)	− 0.02	− 0.03	− 0.01	CT lung involvement reflects objective severity of respiratory disease
Dyspnea	− 0.01	− 0.02	− 0.01	Dyspnea is a clinically relevant symptom indicating respiratory distress
Hypertension	− 0.01	− 0.02	0.00	Hypertension is a known risk factor for severe COVID‑19 outcomes
Cardiovascular disease	− 0.01	− 0.02	0.00	CVD increases vulnerability to complications and hospitalization
WBC count	− 0.005	− 0.01	0.00	WBC variation has a modest impact on risk prediction
Fever	− 0.004	− 0.01	0.00	Fever is common but not highly specific for hospitalization
Cough	− 0.003	− 0.01	0.00	Cough is frequent but a weak predictor of hospitalization
Gender	− 0.002	− 0.005	0.00	Gender shows only modest association with hospitalization risk
Weight/BMI	− 0.001	− 0.005	0.00	BMI contributes minimally in this dataset despite known associations
Myalgia	− 0.001	− 0.003	0.00	Myalgia is nonspecific and has little predictive value
Headache	0.000	0.00	0.00	Headache showed no measurable influence on model performance
Diarrhea	0.000	0.00	0.00	Gastrointestinal symptoms had minimal predictive contribution
Chronic kidney disease	0.000	0.00	0.00	CKD did not show significant impact within this dataset
PCR result	0.000	0.00	0.00	PCR positivity is an entry criterion, not a severity predictor
Platelet count	0.000	0.00	0.00	Platelet count had minimal influence on model discrimination
Hemoglobin	0.000	0.00	0.00	Hemoglobin levels did not directly affect hospitalization prediction
Systolic blood pressure	0.000	0.00	0.00	Systolic BP showed no distinct predictive effect in this dataset
Symptom severity composite	0.000	0.00	0.00	Combined symptom measures did not improve predictive power
Comorbidity count	0.000	0.00	0.00	Total comorbidity count had limited predictive contribution
Other lab feature	0.000	0.00	0.00	Additional laboratory markers did not significantly affect prediction

**Fig. 8:**
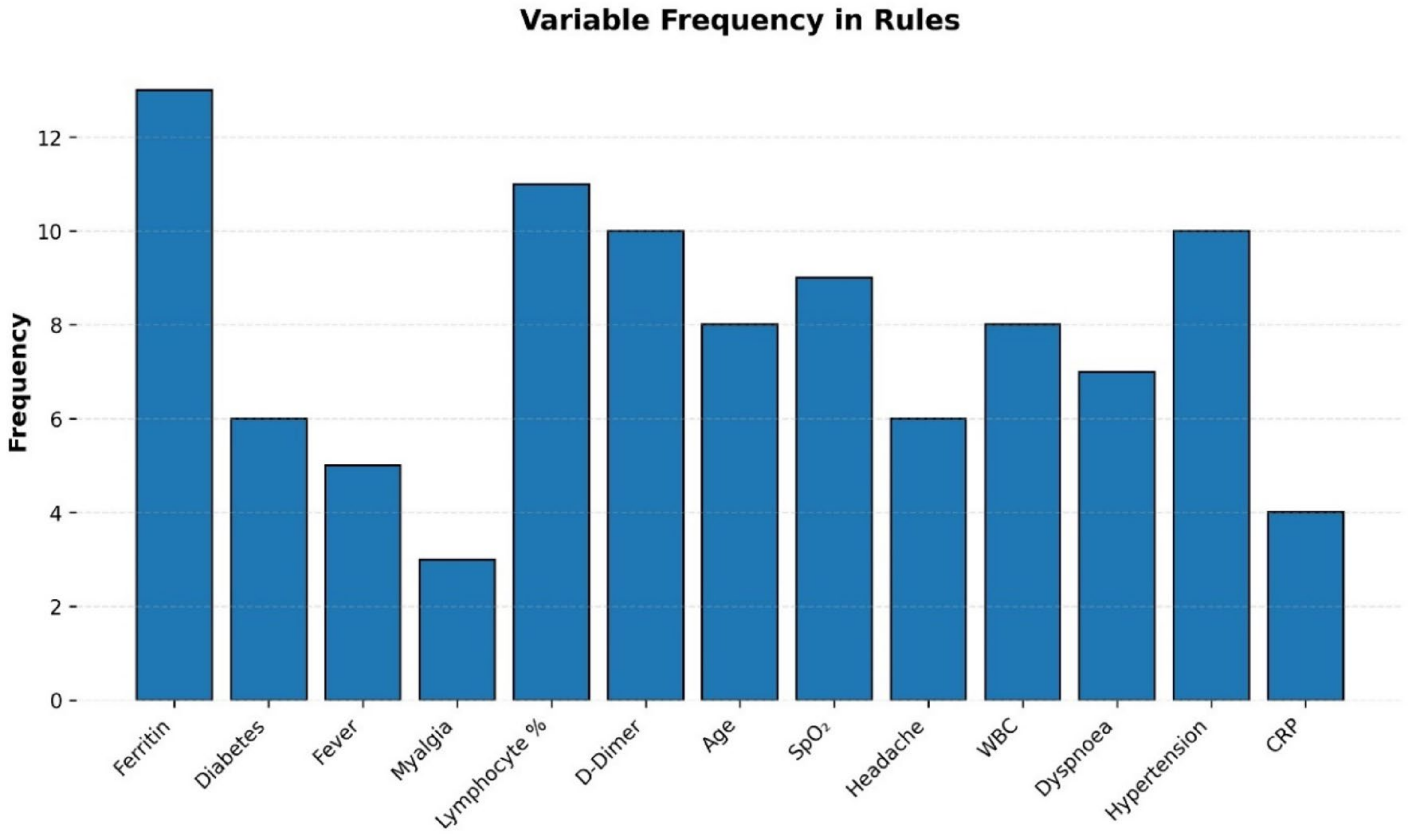
Frequency histogram of clinical features across all cases.

**Fig. 9:**
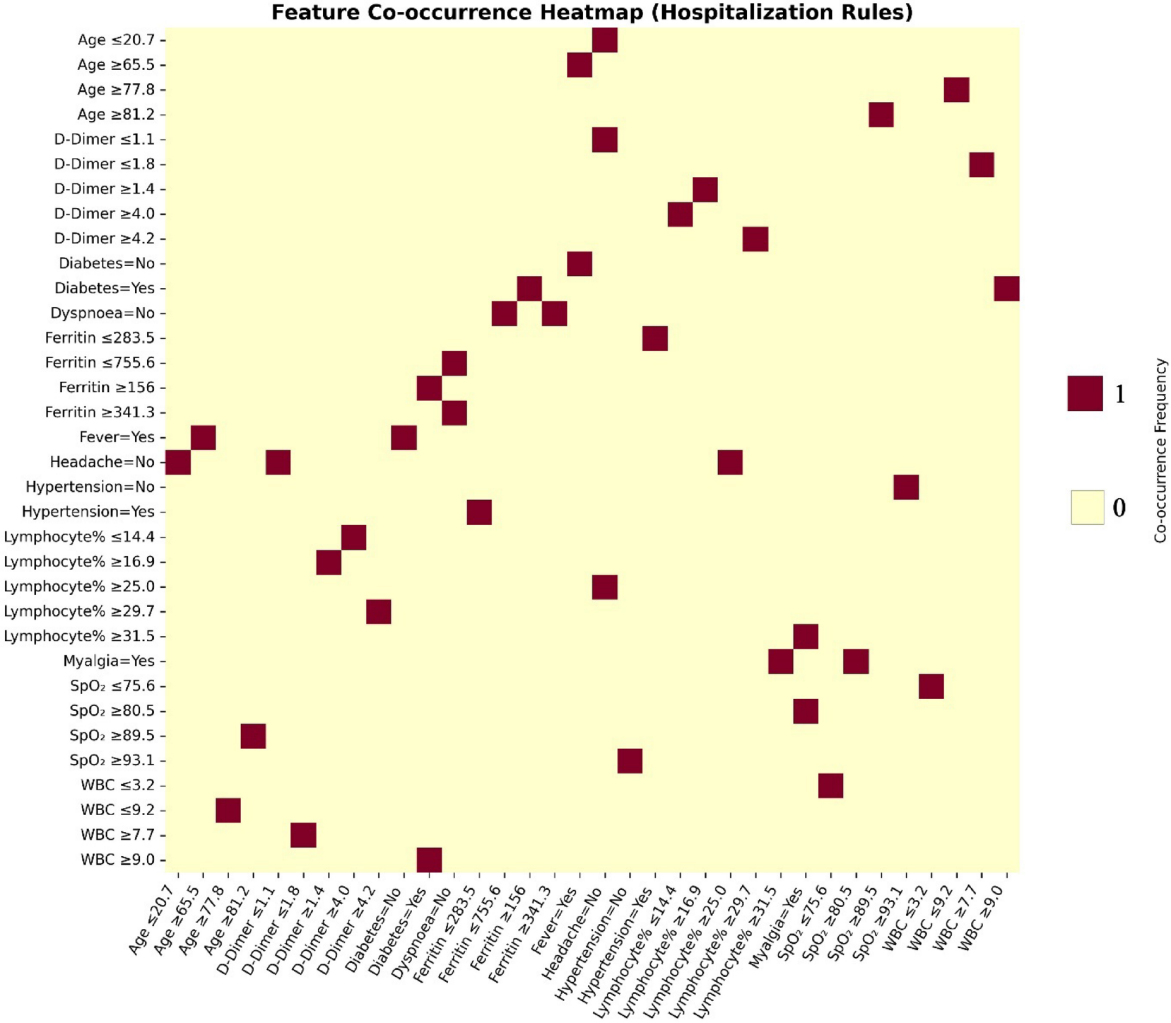
Feature co-occurrence matrix for two-condition rules.

**Fig. 10:**
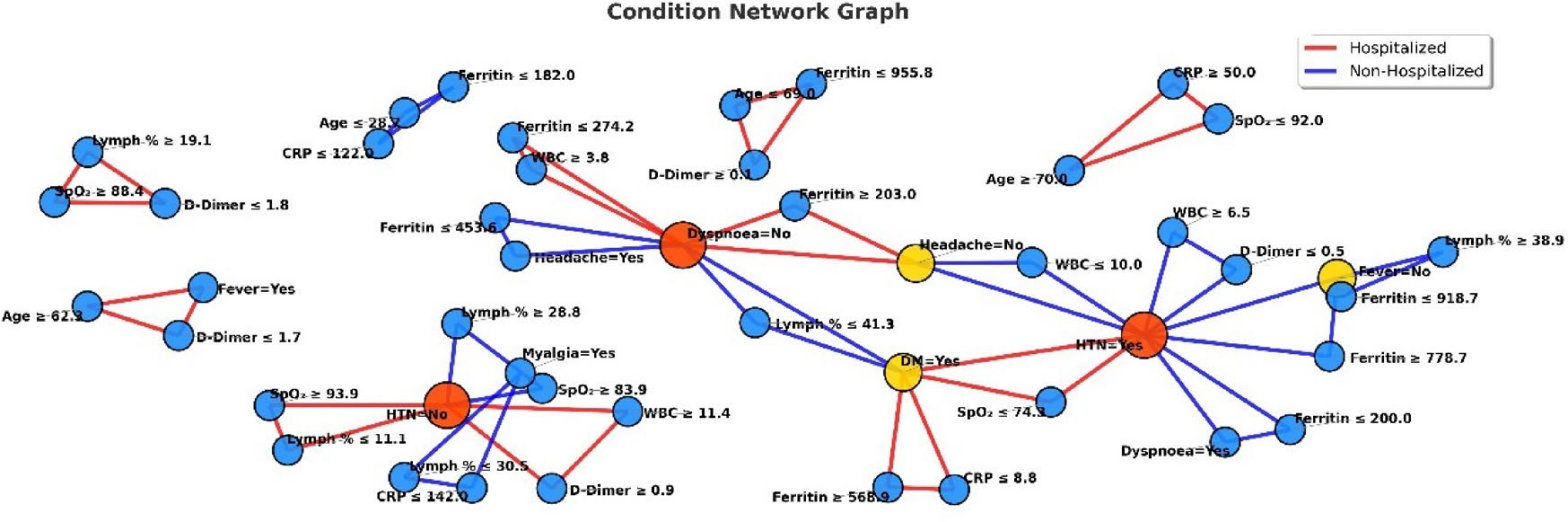
Graph for three-condition rules.

Feature-level analyses are shown in Figs. 8, 9 and 10. The frequency histogram in Fig. 8 highlights ferritin as the most frequently used feature among all rule sets, underscoring its significance as a predictor of hospitalization risk. Conversely, myalgia appeared infrequently, indicating lower discriminative value. The two-and three-condition co-occurrence analyses (Figs. 9 and 10) illustrate how key predictors-such as ferritin, SpO_2_, CRP, D-dimer, and lymphocyte percentage-cluster together to form clinically meaningful rule patterns.

**Fig. 11:**
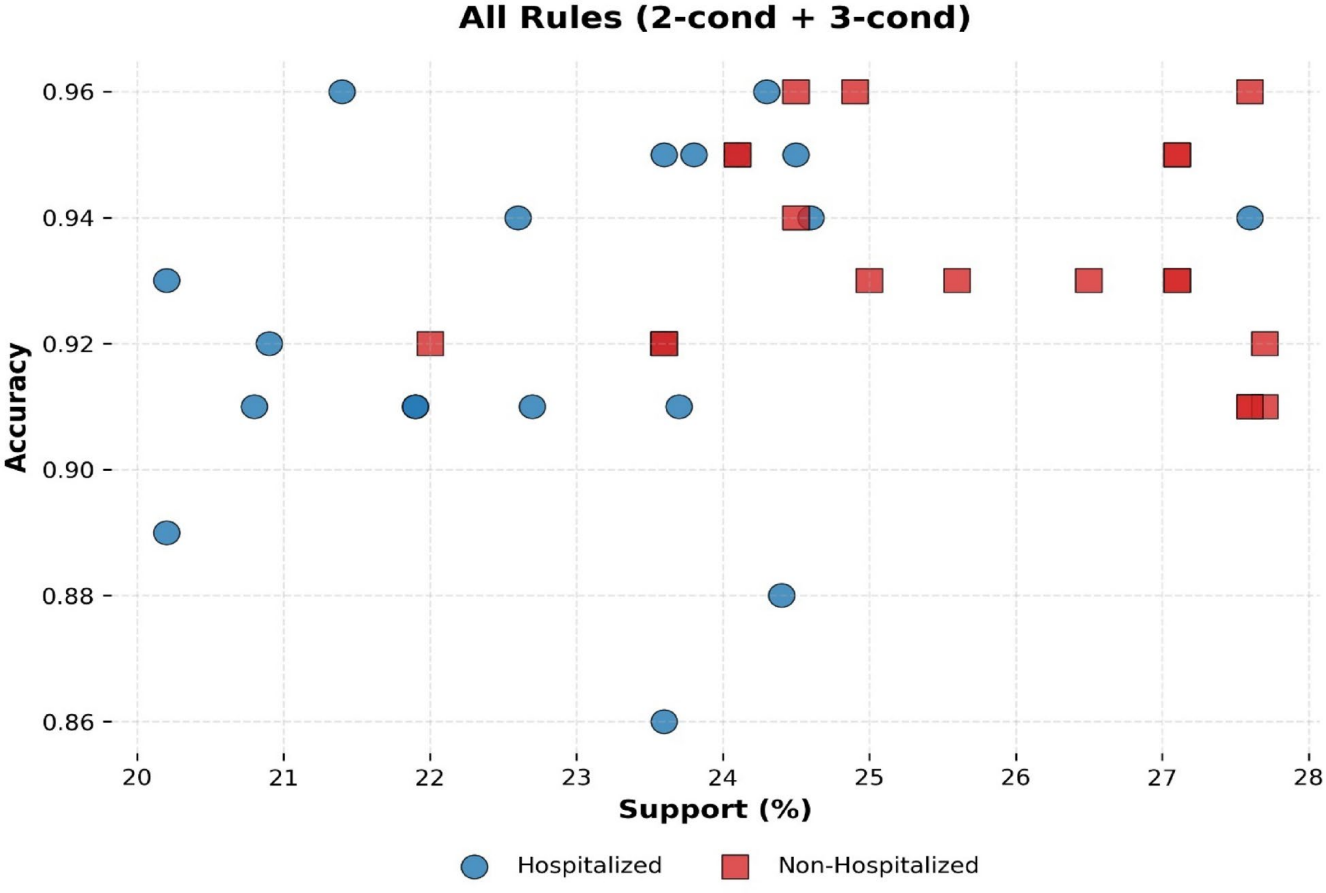
Accuracy versus support of generated rules.

Rule performance is summarized in Fig. 11, which plots Accuracy versus Support across all retained rules. The distribution shows that many rules achieved both high predictive accuracy and adequate coverage, demonstrating that the interpretability component of the model maintains strong technical fidelity to the underlying XGBoost classifier.

Finally, the interpretable decision rules were evaluated using four quality criteria: fidelity, simplicity, interpretability, and support. The rules reproduced the complex model behavior with fidelity above 75%, remained simple by design (only two or three conditions), achieved high expert interpretability scores (CVI ≥ 0.85), and satisfied a minimum Support threshold of 20%. Together, these results confirm that the proposed explainable system is both numerically robust and clinically meaningful for assisting hospitalization decision-making in COVID-19 outpatients.

## Discussion

This study aimed to determine if a system could be designed that combines machine learning models with explainable frameworks to offer accurate predictions while also providing interpretable rationales that physicians would comprehend. Ensuring that physicians can trust and understand the reasoning behind an AI system’s clinical predictions is a fundamental barrier to the practical implementation of AI systems in clinical settings. Consequently, predicting and explaining decisions accurately is a required component of the system. This study provided evidence of interpretable and precise clinical guidelines for predicting hospitalization criteria in patients with COVID-19. Integrated with the XGBoost Algorithm, a black-box model, and a Genetic Algorithm designed to optimize simple rules, a collection of explainable guidelines was created.

One of the main challenges in implementing explainable Algorithms in real-world scenarios is evaluating the rules generated by these models in a clinical context. Previous research has primarily focused on comparing numerical metriscs, such as accuracy, Area Under the Curve (AUC), and F1 score, without sufficient consideration for the comprehensibility and clinical acceptability of the explanations^23,24^. In the field of predicting hospital admissions for COVID-19 patients, research has focused on developing complex models and improving statistical reliability^13,25^. While expert evaluation of the explainable rules generated by evolutionary Algorithms has rarely been conducted, this study evaluates a set of 40 proposed rules by 10 clinical specialists. The validity of the rules was assessed using the conventional Content Validity Index (CVI) specific to this field. This approach increases end users’ confidence and facilitates the use of machine learning in actual clinical settings where precise and unambiguous decision-making is essential. Thus, in contrast to earlier research, this study considers the evaluation of human interpretation as a vital aspect of the proposed system design methodology.

The use of machine learning Algorithms, especially explainable models, to forecast the severity of illnesses and the need for hospitalization in COVID-19 patients has been the subject of several studies in recent years. Jia et al. developed a model using the XGBoost Algorithm, which was enhanced with feature selection techniques and SHAP’s explanatory analysis. The model achieved an AUC of 0.85, accurately identifying patients at risk of clinical deterioration. The researchers emphasized the importance of the model’s interpretability in gaining acceptance among physicians from a clinical perspective^26^. In a related study, Ma et al. predicted impaired lung function (DLCO) in patients recuperating from COVID-19 using an interpretable XGBoost model. The model performed well with an accuracy rate of 78% and an AUC of 0.75. It highlighted important factors, such as C-reactive protein (CRP) levels, lymphocyte percentage, and patient age, in a clear and interpretable manner^27^.

Our explainability framework is inspired by the classical Decision Tree Surrogate approach, which approximates a black-box model using an interpretable tree. However, instead of relying on a single surrogate tree we extend this idea by extracting multiple candidate rules from a Random Forest and refining them through a Genetic Algorithm. This hybrid strategy increases stability, improves fidelity, and yields clearer, clinically meaningful IF–THEN rules. Unlike SHAP or LIME, which provide local feature-attribution explanations for individual cases, our GA-optimized rule-based framework delivers global, human-readable rules that clinicians can apply consistently across patients. This makes the explanations more actionable in real clinical workflows.

The selection of 10 final rules per class was based on an empirical balance between interpretability and predictive stability. Preliminary experiments demonstrated that increasing the number of rules beyond 10 added redundancy, while fewer than 10 reduced clinical coverage and subgroup representativeness. Thus, 10 rules per class provided the optimal trade-off between simplicity and completeness. Rule saturation occurred at around 40 rules.

Benchmark comparisons against Logistic Regression, Random Forest, LightGBM, and a multilayer perceptron neural network demonstrated that XGBoost provided the highest discrimination, calibration, and precision–recall performance. While LightGBM performed competitively, linear models and neural networks showed reduced capability in capturing nonlinear interactions among clinical variables. These results justify the selection of XGBoost as the primary classifier for integration with the explainability framework.

The study demonstrates effective attempts to integrate explainable models with precise predictions. According to their findings, models that clinicians can easily interpret improve the reliability of AI systems and play a key role in clinical applications. Given the complexity of medical decision-making, Algorithms capable of providing interpretable reasoning behind their decisions are of greater scientific value especially during public health crises such as the COVID-19 pandemic. In clinical applications, the goal of this research is to close the gap between interpretability and accuracy. To achieve this, we combine a set of straightforward and understandable rules produced by a Genetic Algorithm with a highly accurate Black-Box model (XGBoost).

One of the key strengths of this study is the design and evaluation of a set of clear, easy-to-understand, and clinically relevant rules for predicting which COVID-19 patients will need to be hospitalized. The rules extracted from the output of the Random Forest model using a Genetic Algorithm and subsequently optimized, provide a logical and interpretable explanation of the relationships among variables such as CRP level, SpO_2_, age, lymphocyte percentage, D-Dimer, and pre-existing conditions relate to the likelihood of hospitalization. For instance, the rules identified as “CRP ≥ 82.1 AND SpO_2_ ≤ 88.6” or “Age ≥ 75.2 AND Dyspnoea = Yes AND Diabetes = Yes”, which are considered as hospitalization predictive indicators in this study, have been verified independently in various research studies. In a study by Jia et al., it was discovered that CRP levels, oxygen saturation, and age were the most influential factors in predicting patient deterioration using the XGBoost model^26^. In a study by Ma et al., it was found that low SpO2 levels, high C-Reactive Protein (CRP) levels, and decreased lymphocyte counts were significant factors associated with long-term respiratory outcomes in recovered patients^27^.

An important aspect of deploying clinical prediction models is ensuring fairness across demographic and clinical subgroups. In this study, subgroup fairness evaluation was performed by comparing model performance across sex, age categories, and key comorbidity groups. The model demonstrated only minor fluctuations in AUC, sensitivity, and specificity among these subgroups, suggesting that it generalizes well without disproportionately underperforming in any vulnerable population. These findings reinforce the reliability of the proposed system for use in diverse patient groups, though future multi-center validation will be necessary to confirm fairness across broader populations.

To further evaluate the robustness of the predictive model, a sensitivity analysis was conducted by iteratively removing each individual feature and observing the resulting change in model performance. Key predictors such as SpO_2_, CRP, age, D-dimer, ferritin, and lymphocyte percentage demonstrated the largest declines in AUC when removed, confirming their central role in hospitalization risk assessment. Conversely, several symptom-level variables such as cough, myalgia, headache, and gastrointestinal symptoms showed negligible impact, aligning with clinical expectations. This sensitivity analysis supports both the internal validity of the model and the clinical plausibility of the extracted rules.

In addition to the items mentioned earlier, several clinical studies have shown a link between pre-existing conditions, such as diabetes and hypertension, and the increased risk of hospitalization. In a systematic review conducted by Noor et al., diabetes, hypertension, and heart disease are significant factors influencing hospitalization and mortality rates in COVID-19 cases^28^. Moreover, ferritin and D-Dimer levels, which were used as complementary indicators alongside other clinical variables in several of the three-condition rules criteria in this study, have also been identified in studies such as Zhou et alas markers of increased inflammation and worsening outcomes in COVID-19^29^. Therefore, not only is the structure of these rules logically and numerically valid, but the evidence from reputable international sources also supports their clinical validity. The alignment between machine learning and clinical data has led to significant advancements in developing interpretable intelligent systems in real-world clinical settings.

The modular architecture of the GA–RF rule extraction process enables rapid re-training on new disease datasets. As long as minimal clinical variables (vital signs + inflammatory markers) are available, the system can regenerate a new rule set within hours, making it adaptable to emerging infectious diseases.

One significant limitation of this study was that it relied on data from just one medical facility in a specific geographic area. While the sample size was moderately appropriate (1278 patients) and included various clinical and paraclinical factors, the results may not be generalizable to populations with different epidemiological, racial, or social characteristics. On the other hand, some influential variables like vaccination history, type of virus strain, and information on the use of specific medications at the time of presentation was either unavailable or insufficiently recorded. Moreover, although the clinical evaluation of the rules was conducted by specialists, the use of complementary statistical methods—such as comparison with expert consensus or User-Perceived Scales (UX)—could have contributed to improving the accuracy of the assessment. Finally, implementation and testing of the proposed system has not been put into practice or evaluated in a clinical setting. Further field studies are needed to determine its evaluation of performance and acceptance in real-world situations.

There are many ways the research can be continued. The recommendations can be included in the CDSS’s hospital software to help determine priority outpatient referrals. By combining this with real-time data and other data sources like continuous vital signs and lung imaging, it can further improve its predictive and explanatory analyses. Conversely, the model developed in this study can be designed to be flexible for other emerging infectious diseases, primarily due to the likely recurrence of such epidemics. The modular design allows for swift changes to be made to the rule sets to keep pace with new variants, changing disease dynamics, or newly added treatments. Particularly for the onset of a pandemic, such algorithms can accurately and rapidly optimize resource distribution to help mitigate the strain on the healthcare system.

### Limitations

This study has several limitations that should be acknowledged. First, the dataset was obtained from a single medical center, which may restrict the generalizability of the findings to broader or more diverse populations. Additionally, although multiple preprocessing steps and expert reviews were implemented to ensure data quality, some potentially influential variables—such as vaccination status, viral strain information, and medication history—were either unavailable or insufficiently documented. Another important limitation is that external validation could not be performed because no comparable post-pandemic datasets were available for retrospective analysis, preventing assessment of the model’s robustness across different healthcare settings. Furthermore, the clinical evaluation of interpretability was conducted by a limited panel of experts, and complementary validation approaches such as inter-rater reliability or user-experience assessments were not included. These factors should be considered when interpreting the results.

### Future work

Future studies should focus on conducting external and multicenter validation to establish the generalizability and robustness of the proposed framework across different clinical environments. Integrating the rule-based explainable component into real-time Clinical Decision Support Systems (CDSS) may further enhance triage workflows and improve early risk stratification. The modular design of the RF–GA rule extraction process also enables rapid adaptation of the system to emerging infectious diseases, allowing the generation of new rule sets as soon as updated clinical data become available. Additionally, incorporating richer data streams—such as continuous vital-sign monitoring, longitudinal laboratory profiles, and imaging-derived biomarkers—may further strengthen predictive performance and interpretability. Finally, future research could explore automated fairness optimization and model simplification strategies to ensure equitable and efficient deployment in real-world clinical settings.

## Conclusion

This research introduced a Framework that combines machine learning Algorithms, specifically XGBoost, with explainable Algorithms based on rule optimization using Genetic Algorithms. The system was developed and evaluated to predict the need for hospitalization in patients with COVID-19. The XGBoost model, known for its strong statistical performance, served as the predictive engine. In addition, a set of 40 clear, easily understandable, and validated clinical rules identified by experts was incorporated as the system’s explainability module’s output. The clinical evaluation of the extracted rules, involving 10 domain experts and based on the Content Validity Index (CVI), confirmed the reliability and relevance of the model’s decision-making logic in real-world clinical contexts. This Two-way approach, combining numerical accuracy with interpretative clarity, demonstrated the potential of machine learning Algorithms as valuable tools for medical decision-making. In conclusion, the proposed framework in this study may serve as a practical model for the design of explainable systems in future public health crises.

## Data Availability

The datasets generated and/or analysed during the current study are available from the corresponding author on reasonable request.
